# Leonardo da Vinci’s “Last Supper”: a case study to evaluate the influence of visitors on the Museum preservation systems

**DOI:** 10.1007/s11356-021-13741-9

**Published:** 2021-04-04

**Authors:** Oriana Motta, Concetta Pironti, Maria Ricciardi, Chiara Rostagno, Ezio Bolzacchini, Luca Ferrero, Raffaele Cucciniello, Antonio Proto

**Affiliations:** 1 Department of Medicine Surgery and Dentistry, University of Salerno, via S. Allende, 84081 Baronissi, Salerno Italy; 2 Ricerca e Progetti Educativi Estero, Direzione Museale Regionale della Lombardia, Palazzo Arese Litta, Corso Magenta, 24 20123 Milan, Italy; 3 GEMMA Center, Department of Earth and Environmental Sciences, University of Milano-Bicocca, Piazza della Scienza 1, 20126 Milan, Italy; 4 Department of Chemistry and Biology, University of Salerno, via Giovanni Paolo II 132, 84084 Fisciano, Salerno Italy

**Keywords:** Leonardo’s Last Supper, Indoor air quality, Human breath, Carbon dioxide isotopic composition, Active and passive sampling, Ammonia, Tourist management

## Abstract

The most important parameter to obtain an appropriate preservation condition of museum environments concerns the indoor air quality. The exposure of artwork and materials to gaseous and particulate pollutants introduced by visitors and either indoor or outdoor sources contributes to their decay. In this work, we evaluated the possible monitoring of the visitors’ influence using the stable carbon isotopic ratio of CO_2_ and the concentration of NH_3_ as a real-time tool. The study was done in the Refectory of Santa Maria delle Grazie (Milan, Italy) which houses one of the most important paintings of Leonardo da Vinci, the Last Supper, and had more than 400,000 visitors in 2019. The results confirmed a good correlation between the presence of tourists inside the museum and the variation of δ^13^C value during the visits and the closure of the museum. The variation of indoor atmospheric δ^13^C was influenced by the presence of visitors in the Refectory and delineates the way done from the entrance to the exit. In the same way, the concentration of NH_3_ was influenced by the presence of visitors and confirmed the role of this one on preservation methodology for indoor air quality in the museum. This new methodology can be used as a supplemental and non-invasive tool to help in calibrating microclimatic conditions through the ventilation rate and air filtration systems in the museum and to manage the number of visitors per turn.

## Introduction

Indoor and outdoor air pollutants could be considered a serious risk parameter for the conservation of artworks in museum environments (Grøntoft 2017; Guerranti et al. 2016). Artworks, including wall paintings, polychrome sculptures, manuscript and canvas paintings, by materials interaction with the environment, go through chemical and physical modifications with a natural and irreversible process of degradation (Drougka et al. 2020). The microclimate conditions of museums are the most common processes of decay, such as wet-dry cycles, thermal shock, and dissolution-crystallisation. The abrupt change of temperature and humidity can produce material stress or fatigue and induce cracking, blistering, scaling, disaggregation, and detachment in wall surfaces as a consequence. Carbon dioxide is one of the most important molecules which is necessary to monitor in indoor environments, because its combination with water vapour may contribute to dissolving alkaline surfaces with the formation of anions and cations that are carried inward where they precipitate as salts (Varas-Muriel et al. 2014). Another result of surface interaction is catalysation of the pigment transformations and discolourations, for example, carbonation processes (Pironti et al. 2017, induce the crystallisation of either cerussite, hydrocerussite (2PbCO_3_ - Pb(OH)_2_), or plumbonacrite (Pb_5_O(OH)_3_(CO_3_)_3_) in presence of Pb pigments (Giustetto et al. 2017). For good indoor air quality, carbon dioxide concentration should not exceed 1000 ppm to also avoid impairment to the health of people in indoor environments, such as “sick building syndrome” (SBS) symptoms (Satish et al. 2012). In literature, many studies considered metabolic emissions from occupants as a primary indoor source for carbon dioxide, and in many cases, its levels in occupied indoor environments were elevated above the local outdoor level (Persily and de Jonge 2017). In a museum, the presence of visitors could be considered the most important source of other gaseous contaminants, such as ammonia. In particular, indoor human source of ammonia was correlated to emission from breath, skin, flatulence, urine, and stool. Schmidt et al. reported an NH_3_ emissions rate from the skin of 20 subjects being 0.3 ng cm^−2^ min^−1^ from the forearms of subjects (Schmidt et al. 2013). Moreover, it is known that nitrite-oxidising bacteria had a high nitrite oxidation activity for transforming ammonia to nitrite. The effect of nitrifying bacteria appeared as a corrosion effect of nitrous and nitric acid. Technical standards define acceptable values of environmental conditions for the conservation of artworks, but case-specific investigations are required to identify the potential degradation factors of each artwork and, as a consequence, the appropriate ranges of microclimatic parameters. Threshold concentrations of gaseous molecules and airborne particulate are required to minimise preservation risks (Braubach and Krzyzanowski 2009; Casati et al. 2015; Mølhave and Krzyzanowski 2003; Settimo et al. 2020; Suess 1992; Tham et al. 2020). In the Refectory of Santa Maria delle Grazie in Milan (Italy), an appropriate heat, ventilation, air conditioning, and cooling system (HVAC) was specifically designed and built for preservation and appropriate conservation of the Leonardo da Vinci “Last Supper”; it is coupled with different types of air filters to remove particulate and gaseous pollutants from the air (air exchange rate from the outdoor around 0.67 h^−1^) (Camuffo and Bernardi 1991; Daher et al. 2011; Gasparini and Christescu 2007).

In the last years, Leonardo da Vinci’s Last Supper was the most studied painting, particularly related to many difficulties with its restorations. Chemical and physical analysis of environments of the Refectory highlighted incompatibilities between restoration and the state of microclimate conditions. In 1983, the Institute of Conservation and Restoration (ICR) started an important project to improve air quality for the best conservation of the Last Supper (ICR 1979). The first study of this project highlighted negative effects of the lighting system such as favouring chemical degradation processes, facilitating the deposition of particulate matter, and other pollutants on the surface of the painting (Brill 1980). Other effects were associated with SO_2_ deposition, produced from the combustion of solid fossil fuels, and considered the most relevant pollutant regarding material deterioration. Thanks to these results, the lighting system was improved and there was a reduction in degradation effects (Gasparini and Christescu 1997; Motta et al. 2014).

In a previous paper, it has been reported that diurnal CO_2_ levels in the Refectory room frequently reached the threshold concentration of 1000 ppm (Gasparini and Stolfi 2014). For this reason, the microclimatic conditions into the Refectory of Santa Maria delle Grazie Church were further improved by physically separating the indoor air (areas 1 and 2 in Fig. 2) at the visitors’ entrance and exit doors, and maintaining a slight overpressure to prevent the diffusion of pollutants from the external urban atmosphere. Temperature and relative humidity are maintained at constant values of 24–25 °C during summer and 20–22 °C in winter, respectively, and RH ~ 50% (Gasparini and Stolfi 2014). Visitors to the museum, even in controlled atmosphere conditions, can be pollutant carriers, and therefore, the number of visitors and the length of the visit are kept strictly limited (30 people for 15 min, from 8:00 AM to 5:00 PM for 6 days/week), and their direction is strictly controlled by providing stops in dedicated rooms (areas 1 and 2 in the map of Fig. 2) to reduce the pollutants they carry. However, this restriction limits the possibility to increase the number of visitors per turn and, consequently, the museum has several hundred unsatisfied visit requests every year.

The identification of the pollution sources and the remediation activities has a significant impact on the organisation of museums. This problem highlighted the requirement of appropriate tools to identify pollutants and their sources as well as to verify the performance of the proactive actions (Ricciardi et al. 2020; Salvatori et al. 2020).

According to the needs of the museum, we proposed the use of isotopic carbon composition of carbon dioxide as an innovative tool to calibrate the ventilation rate of the system based on the number of visitors and the microclimatic conditions. In our work, we analysed the effect of visitors on indoor air quality by the measure of CO_2_ and its isotopic composition, considering that the breath of visitors could introduce new pollutants into the closed environment, besides fine particles (Motta et al. 2009; Pironti et al. 2021).

Carbon dioxide emitted by humans has a characteristic δ^13^C value less than −24.00‰. In some scientific works, the isotopic composition of CO_2_ was used to assess sources at a local scale to discriminate emissions from vehicles from those generated by the biological activity in urban areas (Cucciniello et al. 2012, 2015; Motta et al. 2018; Proto et al. 2014; Zanasi et al. 2006; Pironti et al. 2020b).

**Fig. 1:**
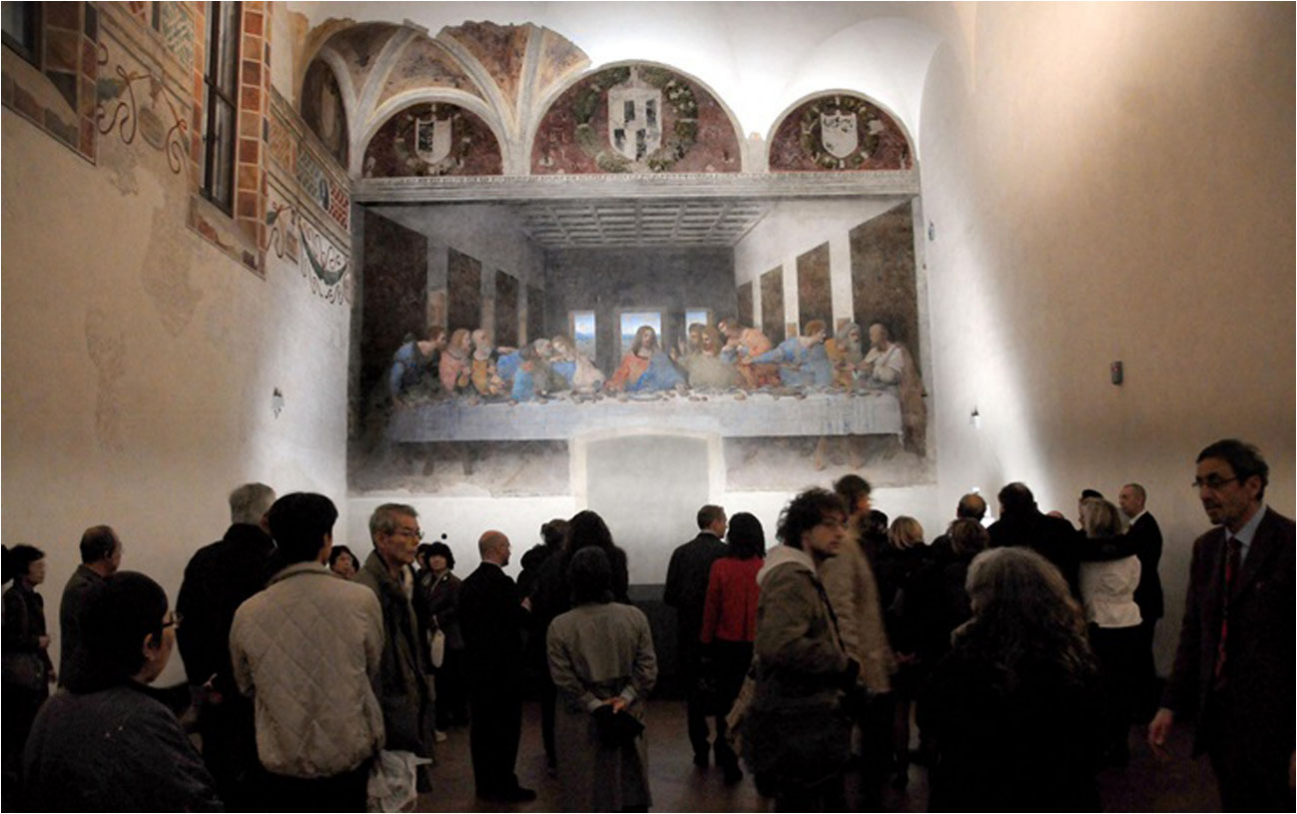
The “Last Supper” with visitors

Generally, isotopic composition was used to set time constraints on processes and manufacturing of objects and to date human samples (e.g., the ^14^C technique). Furthermore, the isotopic composition of metals like Sr and Pb isotopes was useful for tracing the origin of a component or a metal (Alfano et al. 2009; Nord and Billström 2018). In our work, we analysed the possible use of δ^13^C values to refer to atmospheric CO_2_ as a simple tool for the identification and classification of the source of CO_2_ and monitor the influence of tourists on indoor air quality in the Refectory of Santa Maria delle Grazie in Milan, given a possible increase in the number of visitors, thus allowing greater enjoyment of the “Last Supper” (Fig. 1) (Motta et al. 2020). A confirmation of isotopic data was obtained through ammonia air monitoring. In occupied buildings, indoor NH_3_(g) concentrations are typically in the range 15–75 ppb, much higher than outdoor concentrations, and enhancement is consistent with strong emissions from occupants, as previously reported. According to this scientific evidence, in this work, we correlated the ammonia active and passive monitoring to δ^13^C values and the presence of visitors.

## Materials and methods

### Field measurements

The monitoring campaigns were carried out during different seasonal periods for possible various climate characteristics influence (from September to November 2016 and May to July 2017). The visits in the Refectory were organised in groups of about 30 people with a duration of 15 min and a mandatory suggested way. Carbon dioxide for δ^13^C and NH_3_ measurements were collected through active and passive sampling techniques. The passive sampler RING was provided by Aquaria Srl (Milan, Italy), composed of an internal concentric steel cartridge filled with 500.0 mg of a CaO/Ca_12_Al_14_O_33_ 75:25 w/w hydrated sorbent for CO_2_ and with a PE cartridge (with dimensions of 5.8 mm *60 mm) pre-wetted with H_3_PO_4_ as a sorbent for NH_3_.

**Fig. 2:**
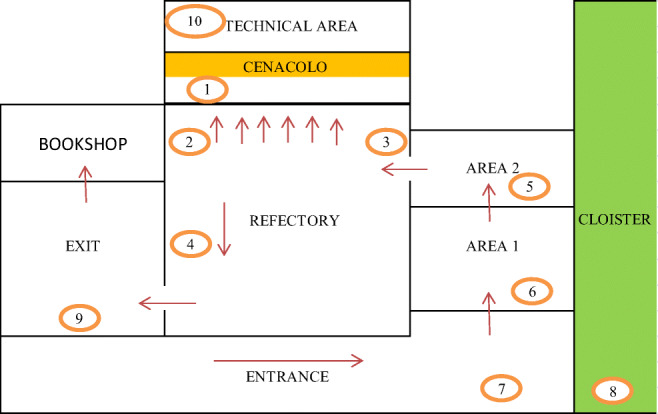
Map of the Museum: the circles represent the position of the passive samplers and arrows suggest the way of visitors

The passive samplers were located at about 2.0 m of height in pre-established positions, as depicted in Fig. 2, for 6 days in triplicates to obtain average concentration values in a week (1 week in September 2016, November 2016, May 2017, July 2017). Circled numbers, showed in Fig. 2, represent the passive samplers along the way of visitors up to the Last Supper painting (entrance, hallway, Refectory with the painting, technical area, bookshop, etc.).

The active monitoring of CO_2_ was conducted on the same days of passive sampling from 7:00 am to 12:00 am in different periods: the first sampling during an interval, the second during the exit, and in the end during the entrance of visitor groups. Each measurement lasted 15 min and samples were collected in triplicate.

For the carbon dioxide active sampling, a tube of ascarite sorbent was used for the first time as a new method, connected to an air pump at a constant flow rate (1 L/min) for 15 min, on the same side of the painting in front of the visitors, thanks to a small hole from the technical room to the Refectory. The sampling flow rate was checked continuously by using a flowmeter.

On the other hand, for ammonia active sampling, a continuous analyser was used based on chemiluminescence principle with NH_3_-NO converter, Ammonia and Nitrogen Oxides Analyzer AC32M-CNH3 by Environnement S.A (111, Boulevard Robespierre, France).

### Preparation of high reactivity calcium oxide substrate for passive CO_2_ sampling

Calcium oxide–based sorbent was prepared as follows: 56.8 g of aluminium nitrate nonahydrate [Al(NO_3_)_3_ 9H_2_O] and 52.4 g of calcium oxide were added into a mixture of distilled water (1.5 L) and 2-propanol (260 mL) so that the weight ratio of calcium oxide to the newly formed mayenite (Ca_12_Al_14_O_33_) would become 75:25 w/w. This solution was stirred for 1 h at 75 °C and successively dried at 120 °C for 18 h before being roasted at 500 °C for 3 h in the air. This method produced a fine and porous powder. After calcination, distilled water was added to the mixture, and the obtained paste was dried at 120 °C for 2 h and then calcined in air at 800 °C for 1.5 h (Cucciniello et al. 2012, 2017; Proto et al. 2014).

All reagents were purchased at reagent grade from Sigma-Aldrich. Before exposure to CO_2_, the sorbent was “activated” by hydrating with water vapour in a closed system for 7 days. Hydration was also necessary to avoid interferences due to variable atmospheric humidity during the exposition.

The crystalline structure of both hydrated and dehydrated sorbents was characterised by XRD analysis.

### Preparation of substrate for active CO_2_ sampling

The active sampler was prepared in the laboratory using Ascarite, 8–20 mesh, as reactive substrate, using two-bed sorbent tubes prepared by introducing 0.8 g and 0.2 g of sorbent into a 10-cm long (i.d of 6.0 mm) glass tube. The second bed sorbent was used as a control septum; both were separated from each other and the environment by glass wool retainers. All reagents were purchased at reagent grade from Sigma-Aldrich and used without other purifications.

### Carbon isotopic ratio analysis

δ^13^C analysis was conducted utilising a HeliFANplus analysed equipped with a single-beam nondispersive infrared industrial photometer. After the exposure at the sampling locations, the sorbent materials were placed into a glass flask and mixed with 2.5 mL of orthophosphoric acid to develop CO_2_ from the carbonates. CO_2_ was then gathered into the aluminised breath bags connected with the inlet ports of the nondispersive infrared (NDIR) spectrometer for sequential measurements. The NDIR device was connected to a computer, which enables the software-guided measurement and calculation of results. Three samples were analysed for each determination.

The carbon isotope ratio was expressed in *δ*‰ relative to V-PDB (Vienna-Pee Dee Belemnite), according to the IUPAC protocol in as follows:

1 $$ {\updelta}^{13}\mathrm{C}=\left[\left({R}_{\mathrm{sample}}-{R}_{\mathrm{standard}}\right)/{R}_{\mathrm{standard}}\right]\times 1000 $$

where *R* is the ratio between the heavier isotope and the lighter one (^13^C/^12^C) (Brand et al. 2014; Pironti et al. 2016, 2020a).

### Ammonia concentration analysis

Ammonia passive samplers were extracted and quantitatively analysed in an aqueous solution using the spectrophotometric determination of indophenol (NIOSH 1994). Ammonia by a reaction with sodium salicylate and chlorine forms a derivative of indophenol, which, in a clearly alkaline environment and the presence of sodium nitroprusside as a catalyst, assumes a green-blue colour, measurable spectrophotometrically at the wavelength of 690 nm. All reagents were purchased at reagent grade from Sigma-Aldrich and used without other purifications. A solution of sodium nitroprusside and sodium salicylate was prepared by dissolving 0.5 g of sodium dihydrate pentacyanonitrosylferrate (III) and 42.5 g of sodium salicylate in 250 mL of distilled water. Sodium dichloroisocyanurate solution 5.8 g/L was prepared by dissolving 0.116 g of sodium dichloroisocyanurate in 20 mL of distilled water. An alkaline solution of sodium citrate (200 g/L) was prepared by dissolving 100 g of trisodium citrate dihydrate and 10 g of sodium hydroxide in 500 mL of distilled water. The oxidising solution was prepared by mixing 80 mL of sodium citrate alkaline solution and 20 mL of sodium dichloroisocyanurate solution.

Samples were extracted with 10 mL of distilled water for 30 min. A total of 5 mL of this solution was shaken with 2 mL of sodium nitroprusside solution and sodium salicylate and 2 mL of oxidising solution. It was necessary to wait 4 h to ensure that the reaction was quantitative before analysing the samples on the UV-vis spectrophotometer. The analyses were carried out using a Cary50 spectrophotometer from Varian.

To calculate the quantity of ammonia present in the air, expressed in μg/m^3^, the following equation was used:

$$ \left[{\mathrm{NH}}_3\right]=78,{54}^{W/t} $$

where *W* is the ammonium (μg) sampling, 78.54 (h/m^3^) is the conversion factor that refers to the flow rate of the sampler, and *t* is the sampling time (h).

## Results and discussion

The results of indoor air monitoring permitted the discrimination of CO_2_ source in the Refectory of Santa Maria delle Grazie and the possibility to delineate a specifically designed programme for visitors.

The values of δ^13^C (‰) obtained were not very different in the areas analysed and were influenced by the visitors inside the museum, reflecting the average value of human breath which is less than −24‰, as reported in the literature (Widory and Javoy 2003). In the first part of our work, we collected the results of passive sampling for CO_2_ and NH_3_ as reported in Table 1.

**Table 1:** δ^13^C (‰) and NH_3_ (μg/m^3^) values obtained from passive sampling in different positions in the Museum during the four campaigns

Sampling period	September (2016)		November (2016)		May (2017)		July (2017)	
Passive sampler position	δ^13^C (‰)	NH_3_ (μg/m^3^)	δ^13^C (‰)	NH_3_ (μg/m^3^)	δ^13^C (‰)	NH_3_ (μg/m^3^)	δ^13^C (‰)	NH_3_ (μg/m^3^)
1	−26.2 ± 0.1	7.1 ± 0.1	−29.7 ± 0.2	5.7 ± 0.1	−26.5 ± 0.1	9.7 ± 0.1	−23.3 ± 0.1	7.2 ± 0.1
2	−24.4 ± 0.1	7.7 ± 0.1	−32.1± 0.1	7.9 ± 0.1	−27.0 ± 0.3	10.9 ± 0.1	−22.0 ± 0.1	9.4 ± 0.1
3	−27.3 ± 0.2	6.2 ± 0.1	−31.2 ± 0.1	7.3 ± 0.2	−30.6 ± 0.1	13.2 ± 0.1	/	8.0 ± 0.1
4	−25.1 ± 0.1	6.0 ± 0.1	−31.0 ± 0.2	6.1 ± 0.1	−34.7 ± 0.1	12.3 ± 0.1	−22.2 ± 0.2	8.4 ± 0.1
5	/	5.0 ± 0.2	−28.3 ± 0.1	3.8 ± 0.1	−33.8 ± 0.2	6.6 ± 0.4	−22.3 ± 0.1	5.7 ± 0.1
6	−22.4 ± 0.2	3.3 ± 0.3	−32.7 ± 0.2	3.6 ± 0.1	−28.8 ± 0.1	7.3 ± 0.1	−17.6 ± 0.1	4.1 ± 0.1
7	−25.5 ± 0.1	5.6 ± 0.2	−37.6 ± 0.1	6.8 ± 0.1	−30.1 ± 0.1	9.5 ± 0.1	−16.1 ± 0.2	13.4 ± 0.1
8	−26.6 ± 0.3	1.9 ± 0.1	−35.6 ± 0.2	1.5 ± 0.1	/	7.4 ± 0.1	−22.3 ± 0.1	4.3 ± 0.1
9	−25.6 ± 0.1	6.2 ± 0.1	−21.0 ± 0.1	6.4±0.1	/	9.5 ± 0.1	−19.2 ± 0.1	12.5 ± 0.1
10	−21.9 ± 0.1	5.6 ± 0.1	−20.4 ± 0.1	6.2±0.1	−20.7 ± 0.1	8.9 ± 0.1	−12.0 ± 0.1	8.2 ± 0.1

The error was expressed as standard error of mean

/ Low concentration of CO_2_ for isotopic analysis

The δ^13^C (‰) values measured in the technical area (10) were higher during the four monitoring campaigns, passing from −21.9 to −12.0‰ because this room was occasionally used by the technical staff to control the diagnostic equipment. This room opens onto the external courtyard of the bookshop, and therefore, the δ^13^C (‰) values found are consistent with that of the external environment. The different isotopic composition during fall, summer, and winter could represent an additional indication for the presence of other pollutants such as ammonia, PM, BTEX, and SO_2._

During the sampling of July, we found different results with respect to the previous months, with values generally higher and closer to the external isotopic composition of CO_2_. These results could be explained by considering that some of the windows on the cloister and the courtyard were opened to the external area to mitigate the high summer temperatures, and the isotopic values increased, looking alike values similar to the external carbon dioxide. This result was also confirmed by a previous work, which analysed the correlation between exposure to indoor particulate matter (PM) and damage to cultural assets and revealed a seasonal average indoor-to-outdoor mass ratios with a maximum value in summer. The authors have also noted that the indoor concentrations of PM were substantially lower than those outdoors and attributed it to the efficacy of the HVAC system in removing infiltrating outdoor pollutants (Daher et al. 2011).

Ammonia passive monitoring shows different concentrations based on the sampler position: in the entrance, the concentration of ammonia was lower compared to other sampling points such as in the Refectory, where all four samplers captured similar ammonia quantities.

In general, the range of concentrations monitored over the 3 months is quite comparable: in May, 6.6 ≤ [NH_3_] ≤ 13.2 μg/m^3^; in July, 4.1≤ [NH_3_] ≤ 13.4 μg/m^3^; and in October and November 1.5 ≤ [NH_3_] ≤ 7.9 μg/m^3^. These results confirmed the influence of the visitors on air quality according to the variation of the concentrations as a function of the position of the sampler.

**Fig. 3:**
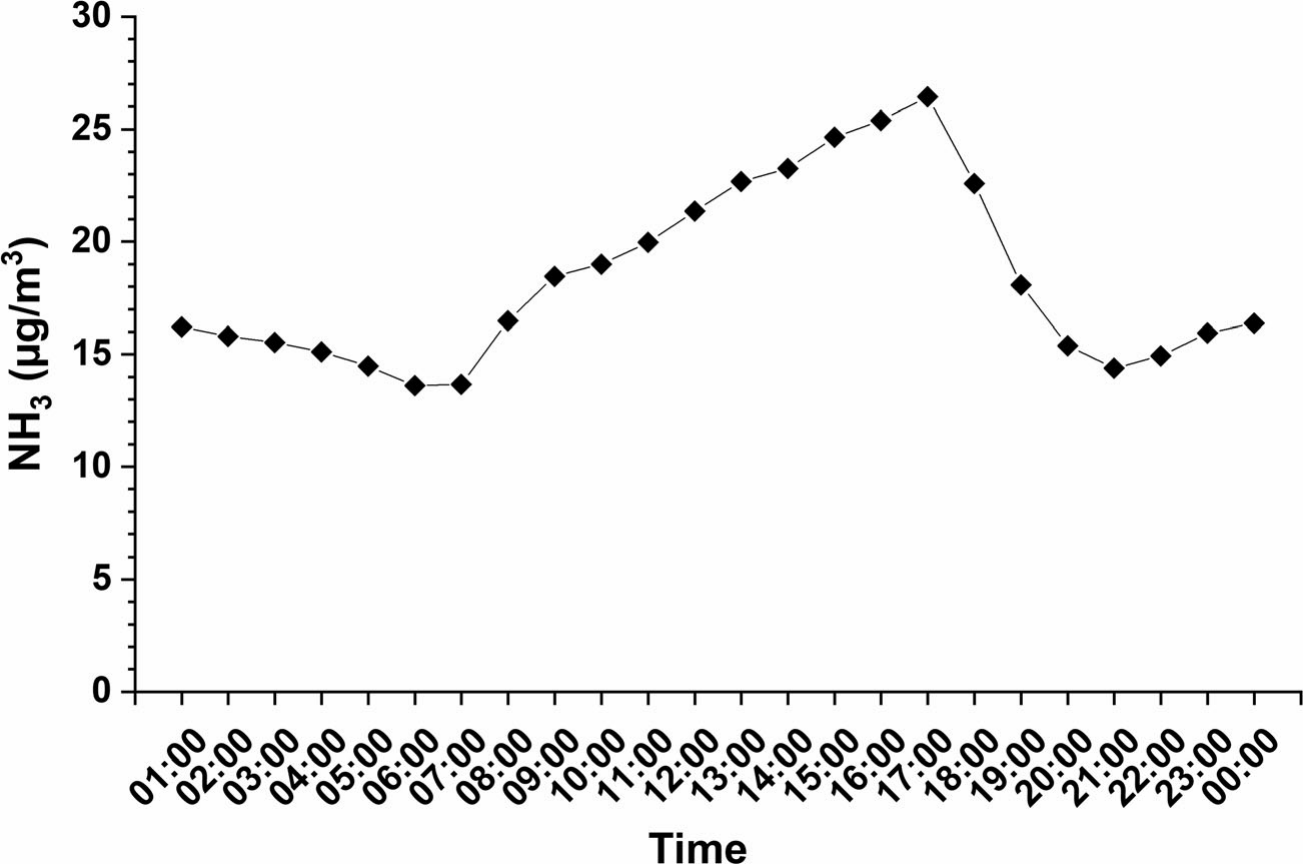
Concentration of NH_3_ obtained by active sampling in the Refectory, from opening to the closure of museum with Ammonia and Nitrogen Oxides Analyzer AC32M-CNH_3_ by Environnement SA

In Fig. 3, the active monitoring of ammonia is shown from 1:00 in the morning to 00:00 PM. The concentration increased at 8:00 AM, corresponding to the opening of the museum (8:15), and reached a maximum value at 5:00 PM. The ammonia concentration decreased during the closing time of the Museum with values comparable and almost constant over the time interval of closure (from 1:00 to 8:00 AM and from 8:00 to 00:00 PM) without tourists or personnel.

**Fig. 4:**
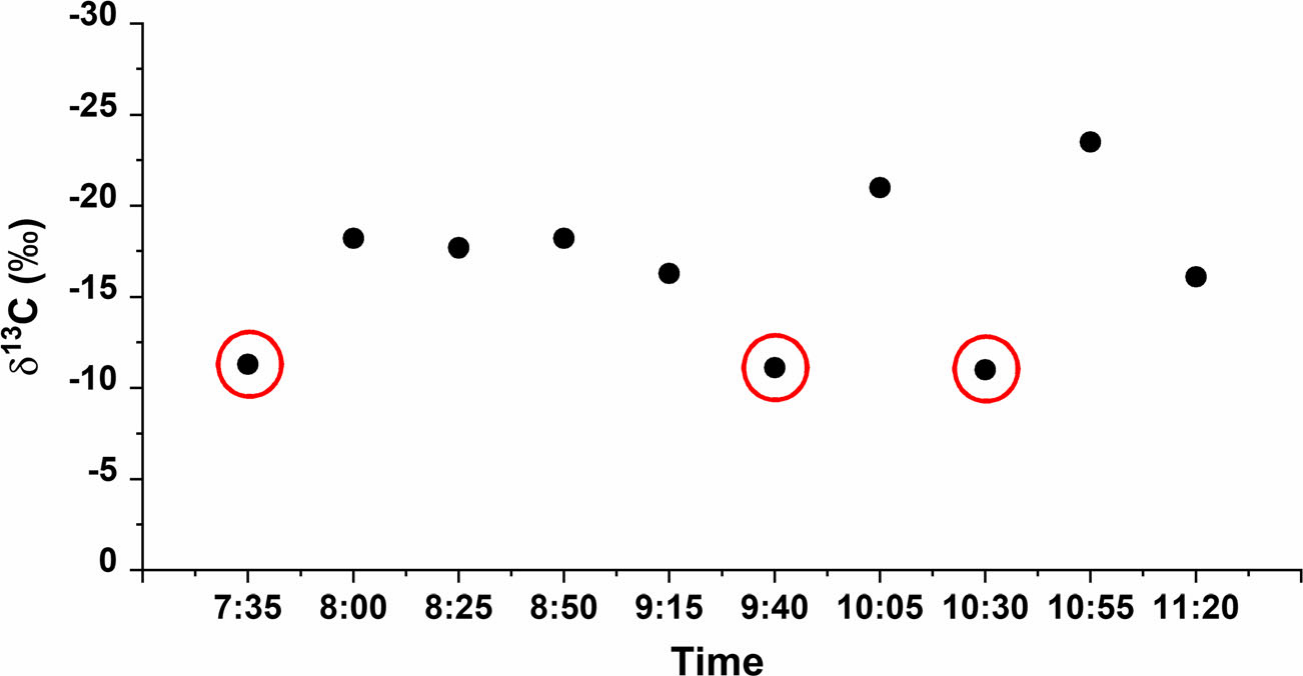
Stable carbon isotopic ratio δ^13^C (‰) obtained by active sampling in the Refectory, red circles correspond to sampling before the opening of the museum

Interestingly, also the results obtained by the active sampling of carbon dioxide confirmed that the carbon isotopic composition of CO_2_ collected in the Refectory were strictly related to visitors. The sampling was carried out in September 2016, starting early in the morning, when the museum was still closed and repeatedly during the visit of different groups of visitors. The results are shown in Fig. 4.

Early in the morning, when the museum was closed, the δ^13^C value measured was −11.3‰. Successively the presence of visitors led the isotopic value to decrease. The points with the red circles correspond to sampling performed before the opening of the museum and in correspondence to the change of the groups of visitors. In this way, we captured the moment when a group was already near the exit, away from the fresco, and the new group was still confined to area 2. It can be observed that during the change of groups of visitors, thanks to the HVAC system, the air was efficiently exchanged leading to a changing in delta values of CO_2_.

Overall, we observed an average of the δ^13^C value of this zone, detected by active samplers (−16.4‰), higher than the average value of −26.4‰ detected with the passive samplers positioned in different parts of the Refectory room. This means that close to the painting, the effects of the ventilation system and the air replacement are very efficient, whereas in different positions, the CO_2_ reflects, on average, the human presence in the room. Active sampling was carried out only in September because the purpose was to detect the change in the carbon isotopic composition of CO_2_ at a specific time of day in the presence/absence of visitors. In fact, due to the higher average frequency of visits during active than passive measurements, the results are not a direct comparison of isotopic value at the location, but even more show the efficient ventilation close to the painting. In literature, a previous work studied the correlation of isotopic carbon composition with environmental pollution in two different archaeological places Fruscione Palace and S. Pietro a Corte in Salerno, Italy. In this work, indoor air quality was influenced by pollutant concentration in the city during many social and cultural events, demonstrating that δ^13^C variation could be a valid tool and non-invasive marker to monitor environmental pollution of museums and cultural heritage. In our study, we can analyse the important effects of visitors on the air quality of a weak environment such as the Refectory of Santa Maria delle Grazie. In particular, the active methodology utilised permits us to obtain information about contaminant concentration at one point in time, while the passive sampling methodology gives contaminant concentration as an average over the whole deployment period.

## Conclusions

In this work, we looked for CO_2_ isotopic composition and ammonia concentration that can be linked to the presence of visitors in the museum of Santa Maria delle Grazie. During the monitoring campaigns, there was clear evidence of the variation of the isotopic composition of CO_2_ with the presence of visitors. The value of the isotopic change found with the passive samplers in the Refectory was, on average, determined by the visitors. The most important results were associated with active sampling that showed the variation of δ^13^C value in the presence/absence of visitors in the Refectory. Early in the morning, δ^13^C value was −11.3‰, very close to the stable isotope value of carbon dioxide in the urban environments, while during a visit of 30 people, the δ^13^C decreased to values associated with their presence. This observation indicates that the air exchange system is very effective near the painting. Therefore, according to the results of this study, the number of visitors and the length of the visit must be strictly limited and controlled to reduce transportation and accumulation of the pollutants in the Refectory. This correlation revealed that the δ^13^C value could be used as a robust and non-invasive marker for real-time evaluation of the air condition systems in the museums, and its monitoring could suggest a strategy for the preventive conservation of artworks and manage the number of visitors per turn.

The indoor air quality correlation with the number of visitors was also confirmed by ammonia monitoring: the passive samplers showed an evident variation of concentrations according to the position of the sampler. The data obtained by active monitoring, positioned in the Refectory behind the painting, highlighted average concentrations of the pollutant at each hour of the day during the week of sampling, and from these results, it was possible to draw a graph that described the trend of ammonia concentration during the day depending on the presence of visitors. The pollutant concentration grows in correspondence to the opening of the museum and has a maximum concentration value at 5:00 PM, while decreases at the closing time. This is clear evidence that ammonia present in the museum can be associated mostly with an anthropogenic source.

This work is also a preliminary study to understand if isotope carbon value could be used to facilitate chemical identification of other pollutants, such as particulate, ammonia, and sulphur dioxide, to reduce the damage on fine artworks and architectural and archaeological heritage, suggesting new and correct procedures of preservation such as rationalisation of entrances, implementation of air filtration systems, and a coherent environmental and architectural conservation approach.
